# New Synthetic Nitro-Pyrrolomycins as Promising Antibacterial and Anticancer Agents

**DOI:** 10.3390/antibiotics9060292

**Published:** 2020-05-30

**Authors:** Maria Valeria Raimondi, Alessandro Presentato, Giovanna Li Petri, Miriam Buttacavoli, Agnese Ribaudo, Viviana De Caro, Rosa Alduina, Patrizia Cancemi

**Affiliations:** 1 Department of Biological, Chemical and Pharmaceutical Sciences and Technologies (STEBICEF), University of Palermo, via Archirafi 32, 90123 Palermo, Italy; mariavaleria.raimondi@unipa.it (M.V.R.); agnese.ribaudo@community.unipa.it (A.R.); viviana.decaro@unipa.it (V.D.C.); 2 Department of Biological, Chemical and Pharmaceutical Sciences and Technologies (STEBICEF), University of Palermo, viale delle Scienze, Building 16, 90128 Palermo, Italy; alessandro.presentato@unipa.it (A.P.); miriam.buttacavoli@unipa.it (M.B.); patrizia.cancemi@unipa.it (P.C.); 3 Pharmaceutical Department, Provincial Health Authority (ASP) of Palermo, via Pindemonte 88, 90129 Palermo, Italy

**Keywords:** heterocycles, pyrrolic nucleus, pyrrolomycin, antibacterial activity, *Pseudomonas aeruginosa*, *Staphylococcus aureus*, antitumoral activity, HCT116, MCF 7

## Abstract

Pyrrolomycins (PMs) are polyhalogenated antibiotics known as powerful biologically active compounds, yet featuring high cytotoxicity. The present study reports the antibacterial and antitumoral properties of new chemically synthesized PMs, where the three positions of the pyrrolic nucleus were replaced by nitro groups, aiming to reduce their cytotoxicity while maintaining or even enhancing the biological activity. Indeed, the presence of the nitro substituent in diverse positions of the pyrrole determined an improvement of the minimal bactericidal concentration (MBC) against Gram-positive (i.e., *Staphylococcus aureus*) or -negative (i.e., *Pseudomonas aeruginosa*) pathogen strains as compared to the natural PM-C. Moreover, some new nitro-PMs were as active as or more than PM-C in inhibiting the proliferation of colon (HCT116) and breast (MCF 7) cancer cell lines and were less toxic towards normal epithelial (hTERT RPE-1) cells. Altogether, our findings contribute to increase the knowledge of the mode of action of these promising molecules and provide a basis for their rationale chemical or biological manipulation.

## 1. Introduction

**Figure 1:**
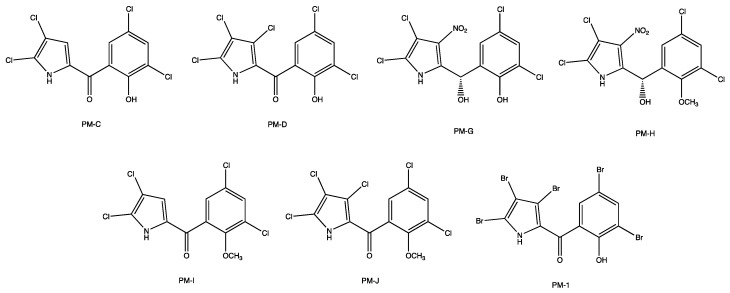
Chemical structure of some natural pyrrolomycins (PMs) and the synthetic one PM **1**.

Pyrrolomycins (PMs) are a family of polyhalogenated antibiotics isolated from the fermentation broth of *Actinosporangium* and *Streptomyces* species [1]. The chemical structure of these metabolites consists of a pyrrole moiety linked to a hydroxyphenyl ring via a one-carbon bridge and characterized by the presence of electron-withdrawing groups (Figure 1). The most remarkable features of these compounds are the pyrrolic nucleus containing three modifiable positions and ionizable groups (i.e., the pyrrole NH and the phenolic OH). Representative examples of PMs include the natural one, named as **C** [2], and that of chemogenic synthesis, called PM **1** [3,4]. PM-**C** can be defined as a tetrachlorine compound since it features two chlorine atoms on both the pyrrolic and the phenolic moieties; differently, the synthetic PM **1** contains five bromines on its scaffold, and this represents the most active pyrrolomycin against *Staphylococcus aureus* synthesized so far [5].

Pyrrolomycins are recognized as molecules with diverse biological activities; indeed, they show potent antimicrobial and antibiofilm properties mainly against Gram-positive bacteria [5,6,7,8,9,10,11]. Besides, some of them display anthelmintic activity [12], as well as neuro-modulatory [13], immune-modulatory [14], insecticidal [15] and anticancer properties. The latter was demonstrated either alone [10,11,16,17] or in combination with other molecules (i.e., the mTOR inhibitor temsirolimus) [18]. 

However, as sound and promising as PMs properties can appear, their polyhalogenation degree could impair their application for biomedical purposes, since, for example, PM-**C** shows acute toxicity on mice [19]. 

Through chemical manipulation, PMs with less toxic moieties could be achieved for improving, on one hand, PMs pharmacokinetic and decreasing their cytotoxicity on the other. Considering that the biological activity of PMs is strictly dependent on the halogens of the pyrrolic nucleus, their replacement with other less toxic electron-withdrawing moieties (i.e., nitro groups) can represent a valid strategy to obtain safer pyrrolomycins.

This aspect can be addressed through synthetic procedures, such as the Microwave-Assisted Organic Synthesis (MAOS), which cost-effectively allows generating new PMs, introducing desired substituents in specific positions by a target manipulation of these compounds [9]. 

The effect of specific chemical modifications on the biological activity can also be helpful to investigate the mode of action of a compound. PMs are thought to explicate their function by binding sortase A in the Gram-positive strain *S. aureus* [9,20,21]. Recently, PMs, such as PM-**C** and PM-**D**, were shown to be powerful depolarizing membrane agents capable of specifically disturbing the proton gradient and decoupling oxidative phosphorylation by protonophoric action [11]. Although PM-**C** resulted more effective than PM-**D** in depolarizing the membrane potential, the biological activity of PM-**D** on both bacterial and cancer cells was greater, likely for its increased lipophilicity and therefore ability to permeate the membrane [11]. 

**Scheme 1:**
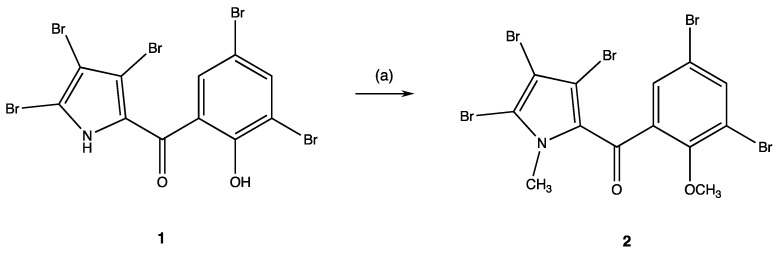
Experimental protocol to obtain compound **2**. *Reagents and reaction condition:* (a) anhydrous dimethylformamide (DMF) as a solvent, anhydrous K_2_CO_3_, magnetic stirring, r.t., 30 min; CH_3_I (10 mmoles excess), magnetic stirring, r.t., 7 days.

Here, taking advantage of previous investigations of some of us [4,5,6,7,8,9] that allowed to synthetically produce halogenated PMs with high yields by MAOS, new PMs were synthesized and their antibacterial and antitumoral activity was assayed. Firstly, the synthesis of the PM **2** (Scheme 1) from the hit compound **1** was carried out to evaluate the importance of the ionizable hydrogens and lipophilicity in the activity of the PMs, replacing, in the PM **2**, the acid hydrogens of the pyrrole NH and phenolic OH groups with methyl ones. 

**Scheme 2:**
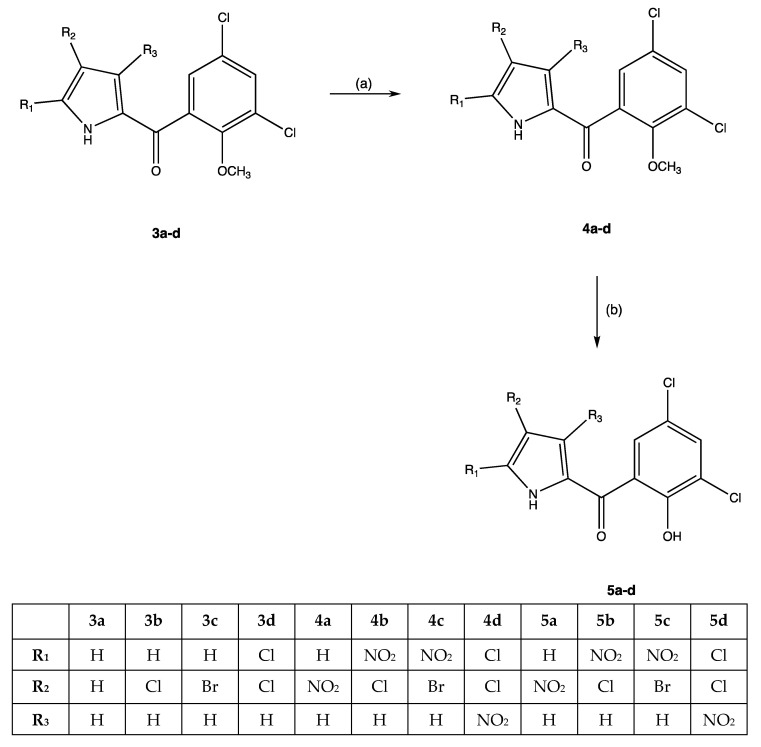
Experimental protocols to obtain compounds **4a**–**d** and **5a**–**d**. *Reagents and reaction conditions:* (a) cold concentrated sulfuric acid as a solvent, equimolar amount of sulfonitric mixture, T = −10 °C, magnetic stirring, 15 min; (b) anhydrous dichloromethane as a solvent, AlCl_3_ (30 mmoles excess), ice-salt bath; magnetic stirring, r.t., overnight; quenching with a cold 5% H_2_SO_4_ solution.

In addition, we introduced the nitro group on the pyrrolic nucleus, generating new nitro-PMs **5a-d** (Scheme 2). The nitro group in different positions led to generate PMs with diverse activity against Gram-positive (*S. aureus*) and -negative (*Pseudomonas aeruginosa*) pathogen strains compared to the PM-**C** and compound **1**. Besides, the nitro-PMs displayed antitumoral activity towards colon (HCT116) and breast (MCF 7) cancer cell lines and, more importantly, featured lower toxicity levels towards normal epithelial (hTERT RPE-1) cells as compared to the natural PM-**C**. We also calculated the ADMET (absorption, distribution, metabolism, excretion, and toxicity) profile of the new PMs **2** and **5a**–**d** to evaluate their pharmacokinetic and toxic properties.

Our results suggested that the biological activity of PMs is due not only to the protonophoric action but also to the steric hindrance and narcotic effect derived from enhanced lipophilicity of PMs in the context of cellular membranes. Moreover, the biological activity of PMs can be modulated by the presence of more electron-withdrawing groups on the pyrrolic nucleus, as well as by the lipophilicity degree, overall representing factors of utmost importance that contribute to decreasing the PM cytotoxicity with respect to those naturally occurring.

## 2. Results and Discussion

### 2.1. Synthesis of new Pyrrolomycins

The methyl-substituted PM **2** was synthesized starting from the pentabromo-pyrrolomycin **1**, which was methylated using excesses of iodomethane (10 mmoles), anhydrous dimethylformamide (DMF) as a solvent, and anhydrous potassium carbonate to generate the salt. After 7 days of magnetic stirring, the organic solvent was removed under vacuum, being, afterward, the PM **2** purified by automated flash chromatography and crystallized (Scheme 1). 

The unsubstituted pyrrolomycin **3a** was obtained with an experimental protocol as previously described [8]. The procedure to obtain pyrrolomycins **3b**,**d** has already been set up through a MAOS approach, using N-bromosuccinimide (NBS) or N-chlorosuccinimide (NCS) as a source of halogens [9]. Since the presence of the acyl group influences the reactivity of the free positions (i.e., R_1_, R_2_, and R_3_) of the pyrrole itself, exploiting this aspect the nitro group was successfully and differently inserted on the pyrrolic nucleus. Furthermore, since the nitration reaction is not selective, to avoid the formation of complex reaction mixtures, it was performed at a low temperature and for a very short time. Consequently, the reaction was carried out using cold concentrated sulfuric acid as a solvent, an equimolar amount of sulfonitric mixture, and working at −10 °C for 15 min under magnetic stirring. Then, the reaction was stopped through a fast work-up process, which involved the use of crushed ice to dilute and cool down the reaction mixture. Once the reaction intermediates were obtained, **4a**,**b**,**d**, these were quickly extracted by using diethyl ether as a solvent and, after evaporation, the crude compounds were purified by crystallization. As for the intermediate of reaction **4c**, which precipitated as a solid in the reaction mixture, it was isolated by filtration and then crystallized. The O-demethylation reaction was performed using a large excess of aluminium chloride as Lewis acid (30 mmoles), in anhydrous dichloromethane as a solvent, cooling the reaction mixture with an ice-salt bath. The reaction mixture was stirred overnight at room temperature, and then a cold 5% sulfuric acid solution was used to decompose the aluminium complex. The nitro-PMs **5a**–**d** were obtained after extraction with diethyl ether and purified by crystallization (Scheme 2).

Analytical and spectroscopic analyses were performed to establish the molecular structures of all new PMs (Figure S1). Specifically, ^1^H-NMR spectra of **4a-d** showed (i) a singlet attributable to the methoxy group in the range of 3.71–3.73 δ, (ii) signals in the range of 7.02–8.27 δ for the aromatic protons, and (iii) a broad singlet, exchangeable with D_2_O, for the pyrrolic NH between 13.25 and 14.72 δ. The presence of the pyrrolic NH was also confirmed by IR spectra that showed broadband in the range of 3174–3264 cm^−1^. 

Similar signals were present in the ^1^H-NMR spectra of PMs **5a-d** for both aromatic protons and the pyrrolic NH; here, a broad singlet related to phenolic OH, exchangeable with D_2_O, in the range of 10.21-11.27 δ was detected instead of the methoxy group. The presence of the phenolic OH was also confirmed by IR spectra that showed broadband in the range of 3309–3585 cm^−1^. In the ^1^H-NMR spectra of the PM **2**, two singlets related to NCH_3_ and OCH_3_ groups are present (3.73 and 3.92 δ, respectively), instead of the pyrrolic NH and phenolic OH. ^13^C-NMR and elemental analysis confirmed the structures.

### 2.2. Antimicrobial Efficacy of New Pyrrolomycins

Previous reports showed significant differences between the minimal inhibitory concentration (MIC) and the minimal bactericidal concentration (MBC) of pyrrolomycins [5,7,11]. Thus, in this study, we compared the MBC of the new PMs to that of the natural PM-**C**—used as the starting compound for the synthesis of nitro-PMs—and to the synthetic PM **1**, which was the precursor of the PM **2**. The latter was included in the analysis to evaluate the influence of both ionizable hydrogens and hydrophobicity of PMs on biological activity. For the most performing PMs, we reported their MIC (Table S1). 

**Figure 2:**
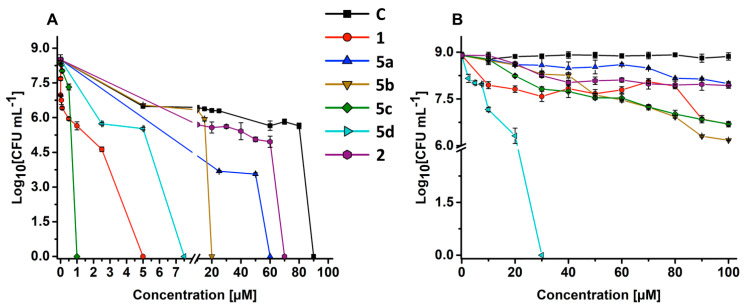
Kill curves of *Staphylococcus aureus* 25923 (**A**) and *Pseudomonas aeruginosa* 10145 (**B**) planktonic cells after challenge with increasing concentrations of PMs for 24 h.

The kill curves of *S. aureus* ATCC 25923 and *P. aeruginosa* ATCC 10145 grown as planktonic cells demonstrated that nitro-PMs **5a**–**d** and **2** displayed a diverse killing effect (Figure 2 and Table 1). Particularly, some PMs needed to reach a certain threshold concentration to explicate their bactericidal action. In this regard, a decrease (~3log) in the number of vital *S. aureus* cells occurred in the presence of increasing concentrations (up to 80 μM) of PM-**C**, while its highest concentration tested (90 μM) determined a bactericidal effect, as highlighted by the total killing of bacterial cells. Nevertheless, the bacteriostatic effect was even more enhanced when *S. aureus* cells were exposed to PM **2**, **5a**, **5b**, and **5d**, since a lesser amount of each PM was needed to roughly achieve the total kill of bacteria. For compound **5c** and **1**, this effect was less evident, likely due to their strongest bactericidal activity (Figure 2A). A reasonable explanation for the diverse mode of action (i.e., bacteriostatic versus bactericidal) elicited by PMs, depending on their concentration, might be ascribed to the lipophilic nature of these compounds. Indeed, hydrophobic compounds with a clogPo/w values higher than 2, and PMs do not represent an exception (Table 2), tend to partition better in the nonpolar context of the phospholipid bilayer, leading to the alteration of the cytoplasmic membrane fluidity and stability through a non-specific mode of action known as narcosis [22,23,24], which can result in severe disturbances of physiological functions, eventually leading to cell death [25,26,27]. To further sustain the importance of PMs’ lipophilicity, the antibacterial activity of the compound **1** was compared to its methoxy analogue (i.e., PM **2**), whose MBC values were 5 μM and 70 μM, respectively. In a previous report, it was shown that the O-methylated PM-**I** and PM-**J** were significantly less active than the non-methylated analogue [11], thus indicating that the ability of PMs to depolarize the membrane is not only attributable to the ionisable OH group present in the phenolic function, but also to that (i.e., NH) of the pyrrolic one. Here, although the (di)methylated PM **2** displayed reduced antimicrobial activity, likely due to the lack of ionisable moieties, it was yet capable of completely inhibiting *S. aureus* growth at an effective concentration that was lower than PM-**C** (Figure 2A). This aspect on one hand emphasized the crucial role played by deprotonable groups in PMs, and on the other hand underlined the not negligible role played by the more hydrophobic PM **2** in the context of the microbial membrane, to which corresponded also a diminished cytotoxicity level with the respect to PM-**C** (Table 1). Indeed, among all the tested PMs, the compound **2** showed a very high hydrophobic component of the solvent accessible surface area (SASA) (saturated carbon and attached hydrogen) and the highest cLogPo/w, which was followed in the order by **1** > **C** > **5d** > **5b** > **5c** > **5a** (Table 2 and Table S2). 

Among the nitro-PMs tested, the **5c**, featuring NO_2_ and bromine substituents in R_1_ and R_2_ positions respectively, was the most proficient in completely inhibiting *S. aureus* cell growth at a concentration as low as 1 µM, therefore being 90 times more active than PM-**C**. The nitro-PM **5b**, yet prone in entirely inhibiting *S. aureus* growth, had higher MBCs (20 µM) as compared to the closely related **5c** (Figure 2A, Table 1), where the main difference relied on the halogen element present in the R_2_ position of the pyrrolic nucleus, being either chlorine (**5b**) or bromine (**5c**). This result showed the importance of the halogen type in the position R_2_. Surprisingly, the new PM **5c** showed a bactericidal activity of 1 µM against *S. aureus*. Since bromine has a lower electronegativity than chlorine, we surmise that the increased activity could be related to a change in the geometric conformation of pyrrolomycins induced by halogen replacement. These results are in contrast with a previous report [28] that demonstrated how the replacement of the chlorine by less electronegative halogens, e.g., bromine and iodine, resulted in a decreased antimicrobial activity. Importantly, the replacement of the chlorine with the bromine in **5c** reduced also its cytotoxicity, as indicated by the selectivity index (SI; Table 1). Among the new nitro-PMs synthesized in this study, the nitro-PM **5c** resulted to be 5-fold more active than the synthetic PM-**1** that contains five bromines on its scaffold, being considered as the best synthetic pyrrolomycin obtained so far against *S. aureus* growth [5,9]. Besides, bacterial SI demonstrated how PM **5c** is 30 times less toxic than PM-**C** (Table 1). 

Upon a change of the NO_2_ group position (R_3_ instead of R_1_) on the pyrrolic nucleus, as well as the presence of chlorine substituents in the R_1_ and R_2_ positions of the nitro-PM **5d**, an increase of its MBC (7.5 μM) was observed. We hypothesize that its activity could be dependent on the synergistic effect derived from the protonophoric action combined with its high lipophilicity, as suggested by the gLogPo/w (Table 2). 

We evaluated whether the replacement of the two chlorine atoms of the PM-**C** pyrrolic ring with a single nitro group in R_2_ (**5a**) could affect the antibacterial activity of pyrrolomycins. Kill curve demonstrated that the nitro-PM **5a** has maintained its ability to inhibit *S. aureus* with an MBC of 60 µM, showing better bactericidal activity than PM-**C**. So far, our findings suggested the importance of the nitro group and its position in the pyrrolic nucleus for biological activity; besides, the lipophilicity also needs to be taken into account as a key factor influencing PM’s potency. We cannot rule out other mechanisms at the membrane or cell wall level. Indeed, in *S. aureus* sortase A was suggested as a specific target of PMs [9].

**Figure 3:**
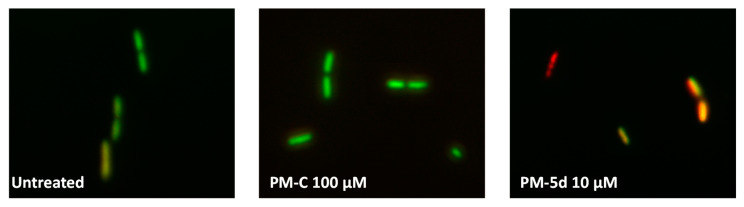
Fluorescence micrographs of *P. aeruginosa* cells treated for 24 h with 100 μM of PM-**C** or 10 μM of PM **5d** after AO/EB double staining. Control represents untreated cells. Green (AO) are viable cells, red (EB) dead cells, and orange vital cells with damaged membranes.

The kill curve of *P. aeruginosa* highlighted how its growth can be completely inhibited by **5d**, which featured an MBC of 30 μM, while the other PMs tested at the highest concentration (100 μM) determined, in the best-case scenario, either 2.7 (i.e., **5b**) or 2.2 (i.e., **5c** and **1**) log reduction in the number of viable colonies forming units (CFUs), as opposed to PM-**C**, which did not display any antimicrobial effect (Figure 2B). Moreover, when *P. aeruginosa* cells challenged with a sub-inhibitory concentration of **5d** (10 μM) were double-stained with both acridine orange (AO; membrane permeable) and ethidium bromide (EB; membrane impermeable) (AO/EB) (Figure 3), cells appeared either orange (indicating that cell membranes were damaged due to the entrance of EB) or red (indicating not viable cells), featuring, the latter, the presence of multiple division septa. Differently, cells treated with 100 μM of PM-**C** appeared green as well as the untreated cells. It is noteworthy to highlight that the main difference between PM-**C** and **5d** relies only on the presence of the nitro group in the position R_3_ of the latter, which is likely responsible for its enhanced electronegativity and, therefore, better biological reactivity over PM-**C**.

To the best of our knowledge, **5d** represents one of the best PMs holding a strong bactericidal activity against a Gram-negative bacterial strain reported so far. Indeed, the MIC of several natural PMs (**C**, **G**, **H**, **I**, **J**) was found to be more than 300 μM against *Escherichia coli* [11,29]. PMs resulted very active against Gram-negative bacteria only when the strains were impaired in the membrane permeability or in the efflux system [11,29,30]. Indeed, MIC values of PMs against *E. coli* strain were strongly reduced only in the case of the ∆*imp* strain, a mutant in the gene encoding for a protein responsible for the membrane permeability and necessary for cell envelope biogenesis, as well as in the case of the ∆*tolC* one*,* a mutant deleted in the gene involved in the biosynthesis of a tripartite efflux pump [29,30]. 

Infections caused by Gram-negative bacteria are usually more difficult to eradicate than those caused by Gram-positive ones because of the presence of an additional outer membrane layer, which protects the cell from the entrance of foreign molecules [31,32,33,34,35,36,37]. Therefore, we assume that the presence of a more electron-withdrawing group on the pyrrolic ring increases the acidity of the hydrogen in N^1^ by an inductive effect, hypothesizing also that the introduction of the nitro group changes PM **5d**’s lipophilicity, therefore facilitating its passage through the outer membrane. Thus, **5d** could represent a lead compound against infections caused by Gram-negative bacterial strains, with a very good selectivity index, which resulted to be higher than the highly cytotoxic and inactive PM-**C** (Table 1).

### 2.3. Antitumoral and Cytotoxic Effects of new Pyrrolomycins

**Figure 4:**
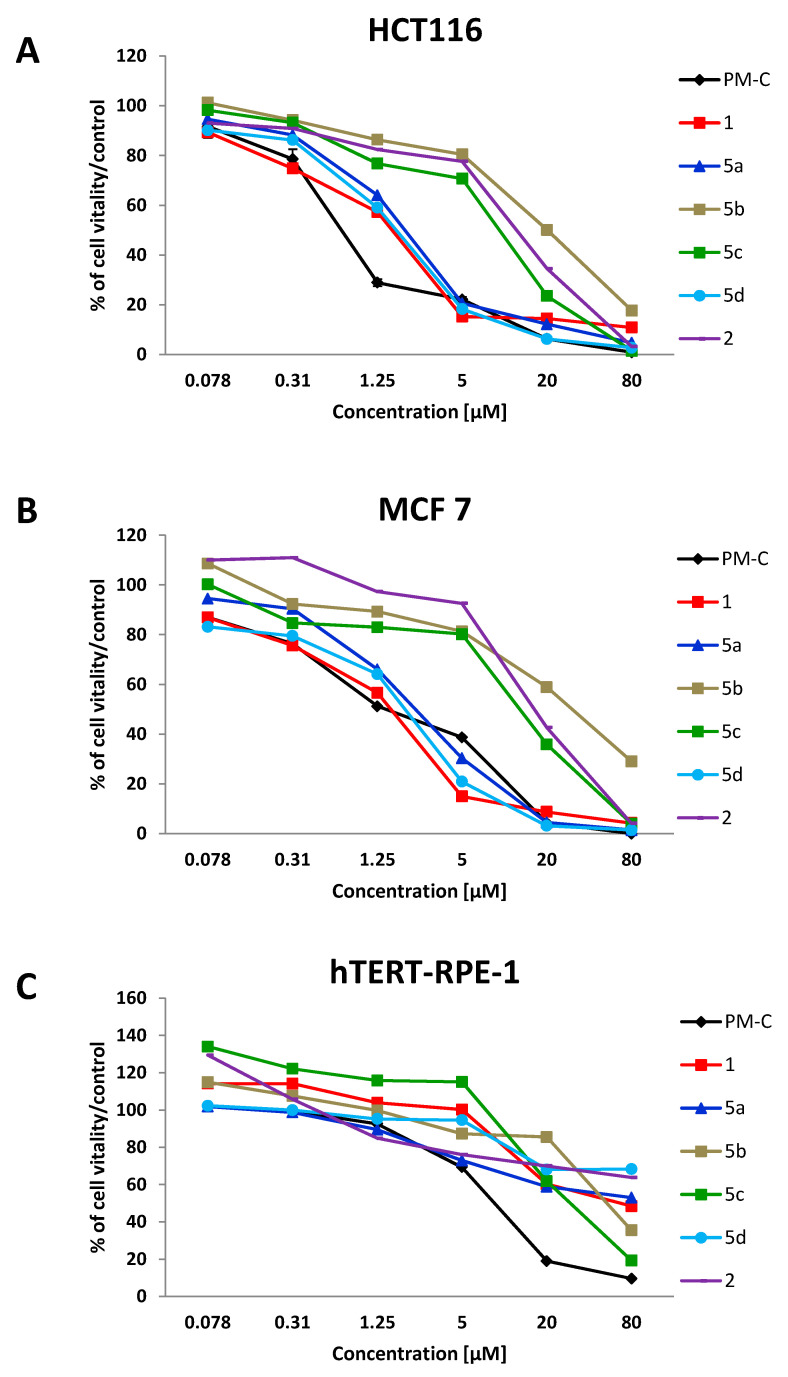
Cytotoxic curves of HCT116 (**A**), MCF 7 (**B**) cancer cells and normal epithelial cell line hTERT-RPE-1 (**C**) after challenge with increasing concentrations of PMs for 48 h.

In addition to the antibacterial activity, the anticancer effect of the PMs was investigated on colon (HCT116) and breast (MCF 7) cancer cell lines, by using the MTT assay (Figure 4A,B). The IC_50_ values, calculated from a dose–response model by fitting sigmoidal curves of the cell percentage inhibition versus the logarithmic concentration of treatments, are reported in Table 1. To evaluate the cytotoxicity of PMs, the MTT assay was performed on the non-tumoral epithelial cell line (hTERT-RPE-1) (Figure 4C and Table 1). Based on the percentage of viable cells after 48 h-challenge, the PMs could be divided in two groups, regardless of the cancer cell line investigated. Indeed, PM-**C**, **1**, **5a,** and **5d** with IC_50_ values ranging from 0.8 ± 0.29 to 2.25 ± 0.35 µM resulted as more potent than the group containing PM **5b**, **5c**, and **2** with IC_50_ values higher than 7 µM (Figure 4A,B and Table 1). Interestingly, the recorded IC_50_ values are within the clinically acceptable concentration [38], suggesting a potential anticancer effect for all the new synthesized PMs. The structure–activity relationship was analysed by comparing the IC_50_ values of selected PMs. The compound **1** showed antitumoral activity comparable to that of the natural one PM-**C**, suggesting the importance of the halogen atoms (both chlorine and bromine) on the pyrrole and the phenyl rings for their biological activity. The effect of deprotonable groups (pyrrolic NH and the phenolic OH) in PMs induced-cytotoxicity was confirmed comparing the IC_50_ values obtained for the compound **1** and the methoxy analogue compound **2**. As expected, the PM **2** (IC_50_ values of 11.13 ± 3.26 µM (HCT116) and 17.25 ± 3.2 µM (MCF 7)) was less active than PM **1** (IC_50_ values of 1.30 ± 0.35 µM (HCT116) and 1.22 ± 0.69 µM (MCF 7)). Interestingly, once again compound **2** retained its biological activity also in the absence of deprotonable groups, suggesting that additional mechanisms besides the protonophoric action could be working in eukaryotic cells. Indeed, similarly to bacterial cells, the biological activity of PMs on eukaryotic cells could be dependent on the lipophilicity. The biological activity of PMs was modulated and improved by chemical changes on the pyrrolic ring. In particular, the introduction of a NO_2_ group in R_3_ (**5d**) or in R_2_ (**5a**) positions maintained the anti-proliferative effects against the cancer cell lines (being IC_50_ of 1.56 µM and 1.90 ± 0.425 for HCT116 and 1.57 ± 0.39 and 2.25 ± 0.35 for MCF 7). On the contrary, the introduction of a NO_2_ group in R_1_ position of **5b** and **5c** PMs reduced their antitumoral activity compared to PM-**C**. The analysis of the cLogPo/w showed a decreased hydrophobicity of both **5b** and **5c** PMs, even if both displayed a high electronegativity, lower only than **5d** that resulted active. Similarly to bacteria, the replacement of the chlorine (**5b**) (IC_50_ of 18.68 ± 3.81 and 28.75 ± 4.21 µM) with the bromine (**5c**) (IC_50_ of 7.64 ± 1.88 and 12.02 ± 2.85 µM) increased the biological activity of the latter. New findings were obtained by assessing the cancer selectivity index (C-SI) (Table 1). The C-SI was low for **5b**, **5c**, PM-**C**, and **2** (with mean values among the two cancer cell lines of 2.5, 8, 8, and 11), while higher C-SI values were found for compounds **1**, **5a**, and **5d** (with mean values of 46, 32 and 143). Thus, the compounds **1**, **5a**, and **5d** are less toxic than PM-C. Based on these results, PM **1**, **5a**, and **5d** were selected for further investigations. 

**Figure 5:**
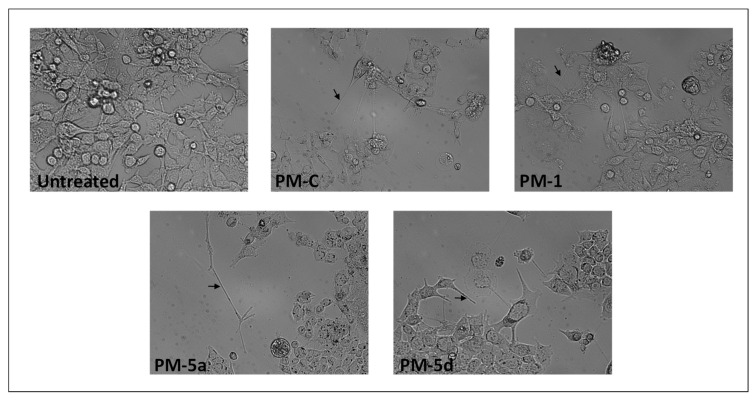
Inverted phase-contrast micrographs of HCT116 cells treated for 24 h with 0.1 µM of selected PMs. Black arrows indicate cell projection, resembling filopodia Magnification 200×.

The morphological evaluation of HCT116 cells treated with sub-inhibitory concentration (0.1 µM) of compounds **1**, **5a**, and **5d** in comparison to PM-**C**, was performed using a phase-contrast inverted microscope (Figure 5). The morphology of HCT116 consisted of a rounded and clumped morphology, while treated cells showed a more fibroblastic and less clumped morphology, became elongated with very long cell projection, resembling filopodia (thin membrane protrusions containing actin microfilaments cross-linked into bundles by actin-bundling proteins). These morphological changes in PMs-treated cells suggested that the impairment of biological membranes together with the cytoskeleton rearrangement could be due to PMs biological activity. In line with this evidence, several authors reported that PMs are able to bind plasma proteins and especially albumin [11,19], therefore, it cannot be excluded that PMs in eukaryotic cells could bind specific proteins.

**Figure 6:**
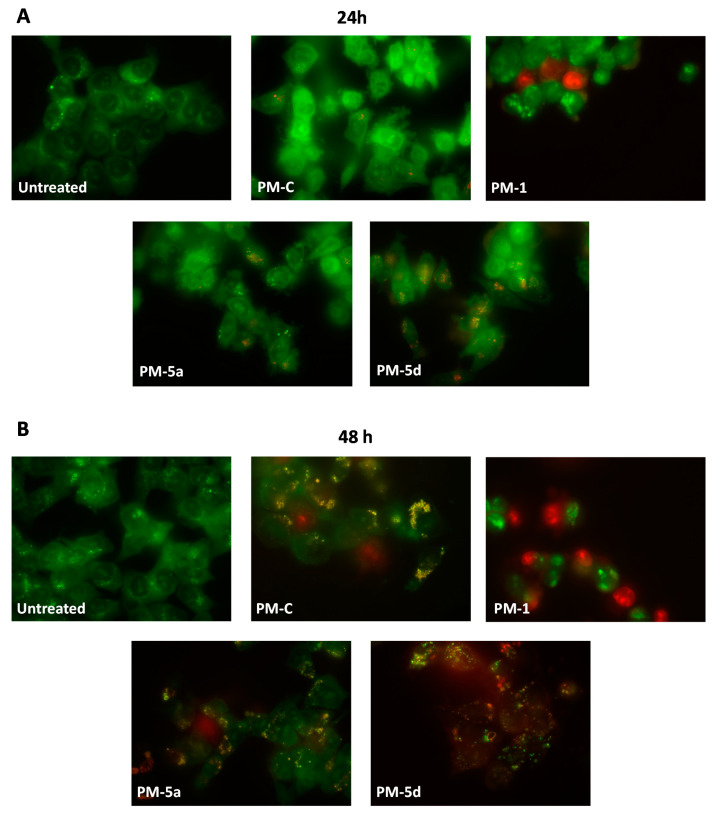
Fluorescence micrographs of HCT116 cells treated for 24 h and 48 h with PMs at IC_50_ after AO/EB double staining. Magnification 630×. Viable cells are uniformly stained in green, without nuclear alteration. Necrotic cells are intensely and uniformly stained in red, showing an increased cytoplasmic volume, while the apoptotic cells are green stained with bright orange or uniformly orange stained with condensed and/or fragmented nuclei.

Double staining with AO/EB of HCT116 cells treated for 24 and 48 h with the IC_50_ (Table 1) of the natural PM-**C**, the compound **1**, **5a**, and **5d** was performed to verify whether the cell death was due to apoptotic induction or unspecific necrosis (Figure 6). As shown in Figure 6, untreated viable cells emitted green fluorescence due to the AO staining of both cytoplasm and nuclei. PM-**C**, **5a**, and **5d**-treated cells exhibited orange fluorescence, indicating a possible induction of apoptosis upon treatment in a time dependent manner. Late apoptosis was evidenced by the presence of reddish orange cells, detected mainly in **5d** treated cells after 48 h, supporting the less cytotoxicity and, therefore, pharmacological suitability of nitro-PMs as compared to the natural PM-**C**. Differently, PM **1** treated cells emitted red fluorescence both at 24 h but with major extent at 48 h, suggesting that cytotoxic effect of the compound **1** is mainly due to necrotic cell death. Our results suggest the nitro-PM **5a** and especially the **5d** could be used as new antitumor agents, for their potency, safety, and type of the induced cell death.

### 2.4. In silico ADMET (Absorption, Distribution, Metabolism, Elimination and Toxicity) Profile of New Pyrrolomycins

Many promising molecules fail to enter in the clinical setting because to their poor pharmacokinetic profile. It is known that a potential drug should be safe and effective, easily administered, preferably orally, easily to absorb and capable of reaching the desired site of action. Moreover, it should not produce toxic metabolites before exerting its pharmacological effect, eliminated easily to avoid the accumulation and consequently undesirable side effects. 

An effective in silico tool to predict the pharmacokinetic profile of drug candidates, in a short time and with lower costs than experimental approaches, is to consider their ADMET (absorption, distribution, metabolism, elimination and toxicity) properties. We used QikProp software (version 6.2, Schrödinger, LLC, New York, NY, 2019) which allowed to compare the properties of each pyrrolomycin with those of 95% of the known drugs. The study permitted to predict 36 properties and the complete results are shown in the Table 2. All the calculated properties of the new synthesized pyrrolomycins, except for the weakly polar solvent accessible surface area (SASA) of **2**, **5b-d** and the cLogP water/gas of **2**, are included within the range of properties of 95% of known drugs indicating that pyrrolomycins **5a**–**d** are promising drug candidates (Table S2). Furthermore, the pharmacokinetic profile of pyrrolomycins was implemented through the prediction of renal and hepatic clearance by pkCSM–pharmacokinetics web tool (Table S3) [39]. Overall, nitro-pyrrolomycins showed a better safety profile than **1** and **C**. 

In Table 2, we reported the most representative ADMET properties of the new pyrrolomycins **2**, **5a**–**d** in comparison with PM-**C** and compound **1**. Although the presence of the nitro group has changed the nitro-pyrrolomycins’ absorption, making them less absorbable orally and less capable of permeating intestinal and blood–brain barriers than PM-**C** and **1**, overall there were no violations of Jorgensen’s rule of three. Furthermore, their lower lipophilia than PM-**C** resulted in a lower ability to bind to plasma proteins, which was considered a pyrrolomycin toxicity factor. The absence of Lipinski’s violations indicated that nitro-pyrrolomycins are considered to be drug-like. 

## 3. Conclusions

In this study, we report on the synthesis of novel PMs showing a low cytotoxicity against normal epithelial cells and endowed with both antibacterial and antitumor activities against Gram-positive or -negative bacteria, as well as colon (HCT-116) and the breast (MCF 7) cancer cell lines. 

Our findings highlight that the biological activity of PMs is influenced by structural changes on the pyrrolic ring. Safe and active PMs were obtained by replacing chlorine with bromine or introducing nitro groups in different positions. Indeed, PM **5c** could be considered as hit antibacterial compound in respect to *S. aureus*, and **5d** against *P. aeruginosa*. Our results showed that the biological activity of PMs is due not only to the protonophoric action but also to the steric hindrance and narcotic effect derived from the lipophilicity of PMs in the context of cellular membranes. Moreover, non-orthodox cell morphology features were evidenced in both bacteria and cancer cells, after their treatment with PMs. It is well known that cytoskeleton is a dynamic network and a potential therapeutic target for new active molecules. Thus, the effect of PMs on cytoskeleton remodelling deserves further investigation. 

In conclusion, PMs are promising molecules not only for their biological activity, but more importantly for their mode of action on the membranes, because the emergence of drug (both antibiotic and antitumoral) resistance is hampered by the lack of a gene-encoded target. 

## 4. Experimental Section

### 4.1. Chemistry

#### 4.1.1. Materials and Instruments

Melting points were determined on a Stuart Melting Point Apparatus SMP30 and are uncorrected. IR spectra were recorded at room temperature in KBr disks with a Perkin Elmer Infrared 137 E spectrometer. ^1^H-NMR (300 MHz) and APT (75 MHz) spectra were recorded with a Bruker AC-E spectrometer at room temperature in DMSO-d_6_, using tetramethylsilane as internal standard; chemical shifts (δ) are expressed as ppm values. Microanalyses (C, H, N) were carried out with an Elemental Vario EL III apparatus and agreed with theoretical values ± 0.4%. Automated flash chromatography was performed with a CombiFlash Rf200 system (TeleDyne ISCO) using Silica gel 60 cartridges (0.040–0.063 mm, 230–400 mesh ASTM). All reactions were monitored by TLC on precoated aluminum sheets 20 × 20 (0.2 mm Kieselgel 60 G F254, Merck), using UV light at 254, 365 nm for visualization. All reagents and solvents were from Aldrich, Merck, Across and Carlo Erba Reagents. ADMET properties were calculated by QikProp (version 6.2, Schrödinger, LLC, New York, NY, 2019) and pkCSM web tool.

#### 4.1.2. Methods

Known compounds **1** and **3a**–**d** were prepared and characterized as previously described [8,9]. 

General procedure for nitration of (1*H*-pyrrol-2-yl)(3,5-dichloro-2-methoxyphenyl)methanones (**3a**–**d**) to obtain compounds **4a**–**d**.

To a magnetically stirred solution of 3.13 mmoles of **3a**–**d** in 20 mL of cold concentrated sulfuric acid (T = −10 °C), an equimolar amount of sulfonitric mixture was added. The reaction mixture was left under stirring for 15 min at −10 °C, and crushed ice was then added. The (4-bromo-5-nitro-1*H*-pyrrol-2-yl)(3,5-dichloro-2-methoxyphenyl)methanone **4c** was isolated by filtration and crystalized from ethanol. 

Compounds **4a**,**b**,**d** were isolated by extraction with diethyl ether (3 × 50 mL). Then, the combined extracts were washed with brine (2 × 30 mL), dried over anhydrous sodium sulfate and evaporated. The crude products were purified by automated flash chromatography using a mixture of ethyl acetate/cyclohexane (*v*/*v* 20/80) as eluent, and then crystalized from ethanol. 

##### (3,5-Dichloro-2-methoxyphenyl)(4-nitro-1*H*-pyrrol-2-yl)methanone (**4a**)

White solid, yield 65%, mp 162–164 °C. ^1^H-NMR: δ 3.73 (s, 3H, OCH_3_), 7.16 (s, 1H, H-3), 7.57 (s, 1H, H-6′), 7.86 (s, 1H, H-4′), 8.27 (s, 1H, H-5), 13.35 (br s, 1H, NH, exc. D_2_O). IR (cm^−1^): 3220 (NH), 1663 (CO). Anal. calc. for C_12_H_8_Cl_2_N_2_O_4_: C, 45.74%; H, 2.56%; N, 8.89%. Found: C, 45.87%; H, 2.73%; N, 8.98%.

##### (4-Chloro-5-nitro-1*H*-pyrrol-2-yl)(3,5-dichloro-2-methoxyphenyl)methanone (**4b**)

White solid, yield 56%, mp 158–160 °C. ^1^H-NMR: δ 3.73 (s, 3H, OCH_3_), 7.02 (s, 1H, H-3), 7.59 (s, 1H, H-6′), 7.90 (s, 1H, H-4′), 13.25 (br s, 1H, NH, exc. D_2_O). IR (cm^−1^): 3230 (NH), 1671 (CO). Anal. calc. for C_12_H_7_Cl_3_N_2_O_4_: C, 41.23%; H, 2.02%; N, 8.01%. Found: C, 41.38%; H, 2.12%; N, 8.11%.

##### (4-Bromo-5-nitro-1*H*-pyrrol-2-yl)(3,5-dichloro-2-methoxyphenyl)methanone (**4c**)

White solid, yield 78%, mp 130–132 °C. ^1^H-NMR: δ 3.71 (s, 3H, OCH_3_), 7.03 (s, 1H, H-3), 7.55 (s, 1H, H-6′), 7.88 (s, 1H, H-4′), 14.72 (br s, 1H, NH, exc. D_2_O). IR (cm^−1^): 3264 (NH), 1668 (CO). Anal. calc. for C_12_H_7_BrCl_2_N_2_O_4_: C, 36.58%; H, 1.79%; N, 7.11%. Found: C, 36.70%; H, 1.92%; N, 7.39%.

##### (3,5-Dichloro-2-methoxyphenyl)(4,5-dichloro-3-nitro-1*H*-pyrrol-2-yl)methanone (**4d**)

White solid, yield 45%, mp 168–170 °C. ^1^H-NMR: δ 3.71 (s, 3H, OCH_3_), 7.56 (s, 1H, H-6′), 7.85 (s, 1H, H-4′), 13.84 (br s, 1H, NH, exc. D_2_O). IR (cm^−1^): 3174 (NH), 1618 (CO). Anal. calc. for C_12_H_6_Cl_4_N_2_O_4_: C, 37.54%; H, 1.58%; N, 7.30%. Found: C, 37.68%; H, 1.71%; N, 7.42%.

##### General Procedure for Preparation of Pyrrolomycins **5a**–**d**

To a magnetically stirred solution of 1 mmol of **4a**–**d** in 20 mL of anhydrous dichloromethane in an ice–salt bath, 30 mmoles of AlCl_3_ were added. The reaction mixture was left under stirring overnight at room temperature. The solution was cautiously decomposed with a cold 5% sulfuric acid solution (30 mL), then 50 mL of diethyl ether were added. The mixture was stirred vigorously for 10 min, the organic layer was separated and the aqueous phase extracted with diethyl ether (2 × 30 mL). The combined extracts were washed with brine until neutrality, dried over anhydrous sodium sulfate, and evaporated. The crude product was crystallized from dichloromethane to give pure pyrrolomycins **5a**–**d**. 

##### (3,5-Dichloro-2-hydroxyphenyl)(4-nitro-1*H*-pyrrol-2-yl)methanone (**5a**)

Yellow solid, yield 90%, mp 184–185 °C. ^1^H-NMR: δ 7.19 (s, 1H, H-3), 7.49 (s, 1H, H-6′), 7.76 (s, 1H, H-4′), 8.27 (s, 1H, H-5), 10.51 (br s, 1H, OH, exc. D_2_O), 13.32 (br s, 1H, NH, exc. D_2_O). ^13^C-NMR: δ 113.68, 123.44, 123.87, 126.56, 128.15, 130.68, 132.39, 137.36, 150.76, 183.27. IR (cm^−1^): 3583 (OH), 3206 (NH), 1618 (CO). Anal. calc. for C_11_H_6_Cl_2_N_2_O_4_: C, 43.88%; H, 2.01%; N, 9.30%. Found: C, 43.97%; H, 2.11%; N, 9.34%.

##### (4-Chloro-5-nitro-1*H*-pyrrol-2-yl)(3,5-dichloro-2-hydroxyphenyl)methanone (**5b**)

Yellow solid, yield 85%, mp 180–182 °C. ^1^H-NMR: δ 7.18 (s, 1H, H-3), 7.60 (s, 1H, H-6′), 7.87 (s, 1H, H-4′), 11.23 (br s, 1H, OH, exc. D_2_O), 13.41 (br s, 1H, NH, exc. D_2_O). ^13^C-NMR: δ 109.13, 124.92, 128.90, 129.19, 129.88, 132.97, 133.05, 141.98, 153.72, 166.39, 182.89.IR (cm^−1^): 3585 (OH), 3348 (NH), 1629 (CO). Anal. calc. for C_11_H_5_Cl_3_N_2_O_4_: C, 39.38%; H, 1.50%; N, 8.35%. Found: C, 39.47%; H, 1.61%; N, 8.47%.

##### (4-Bromo-5-nitro-1*H*-pyrrol-2-yl)(3,5-dichloro-2-hydroxyphenyl)methanone (**5c**)

Yellow solid, yield 52%, mp 175–177 °C. ^1^H-NMR: δ 7.03 (s, 1H, H-3), 7.56 (s, 1H, H-6′), 7.88 (s, 1H, H-4′), 11.27 (br s, 1H, OH, exc. D_2_O), 14.60 (br s, 1H, NH, exc. D_2_O). ^13^C-NMR: δ 98.35, 121.01, 123.10, 124.33, 127.67, 128.97, 133.06, 134.02, 138.57, 152.26, 183.47. IR (cm^−1^): 3534 (OH), 3298 (NH), 1656 (CO). Anal. calc. for C_11_H_5_BrCl_2_N_2_O_4_: C, 34.77%; H, 1.33%; N, 7.37%. Found: C, 34.98%; H, 1.42%; N, 7.44%.

##### (3,5-Dichloro-2-hydroxyphenyl)(4,5-dichloro-3-nitro-1*H*-pyrrol-2-yl)methanone (**5d**)

Yellow solid, yield 85%, mp 180–182 °C. ^1^H-NMR: δ 7.51 (s, 1H, H-6′), 7.90 (s, 1H, H-4′), 10.21 (br s, 1H, OH, exc. D_2_O), 13.60 (br s, 1H, NH, exc. D_2_O). ^13^C-NMR: δ 105.15, 107.20, 111.26, 113.56, 130.03, 130.69, 131.32, 137.15, 151.89, 180.94. IR (cm^−1^): 3568 (OH), 3317 (NH), 1641 (CO). Anal. calc. for C_11_H_4_Cl_4_N_2_O_4_: C, 35.71%; H, 1.09%; N, 7.57%. Found: C, 35.88%; H, 1.15%; N, 7.69%.

##### Synthesis of the (3,5-Dibromo-2-methoxyphenyl)(3,4,5-tribromo-1-methyl-1*H*-pyrrol-2-yl)methanone (**2**)

To a magnetically stirred solution of 1 mmol of pyrrolomycin **1** in 10 mL of anhydrous dimethylformamide (DMF), 30 mmoles of K_2_CO_3_ were added and the suspension was stirred for 30 min at room temperature to generate the salt. Then, 10 mmoles of CH_3_I were added under stirring at room temperature, and the reaction was monitored every 24 h on TLC. After 7 days, it was completed and the solvent was then removed under vacuum. Compound **2** was purified by automated flash chromatography using a mixture of ethyl acetate/acetone (*v*/*v* 50:50) as eluent and then crystalized from ethanol.

##### (3,5-Dibromo-2-methoxyphenyl)(3,4,5-tribromo-1-methyl-1*H*-pyrrol-2-yl)methanone (**2**)

White solid, yield 68%, mp 179–180 °C. ^1^H-NMR: δ 3.73 (s, 3H, NCH_3_), 3.92 (s, 3H, OCH_3_), 7.71 (s, 1H, H-6′), 8.09 (s, 1H, H-4′). ^13^C-NMR: δ 37.86, 62.57, 104.48, 109.17, 116.80, 118.20, 118.65, 130.40, 132.09, 136.30, 138.02, 154.31, 181.30. IR (cm^−1^): 1648 (CO). Anal. calc. for C_13_H_8_Br_5_NO_2_: C, 25.61%; H, 1.32%; N, 2.30%. Found: C, 25.73%; H, 1.43%; N, 2.42%.

#### 4.1.3. In silico ADMET (Absorption, Distribution, Metabolism, Excretion and Toxicity) Calculation

Pharmacokinetic studies to estimate the absorption, distribution, metabolism, excretion and toxicity (ADMET) of pyrrolomycins were performed by using QikProp Schrödinger (LLC, New York, NY, 2019). The molecular structures were designed in 3D and their conformations were optimized with the MMFF94 tool. Finally, the ADMET properties have been calculated with QikProp, running in normal mode, and by pkCSM—pharmacokinetics web tool [39].

### 4.2. Biology

#### 4.2.1. Biocidal and Inhibitory Activity of New Pyrrolomycins

Kill curve assays were performed to test the antimicrobial efficacy of pyrrolomycins (i.e., **1**, **2**, **5a**, **5b**, **5c**, **5d**), as well as that of the natural PM-**C** against the indicator pathogen *Staphylococcus aureus* ATCC 25923 and *Pseudomonas aeruginosa* ATCC 10145 strains. Briefly, a single colony of either *S. aureus* or *P. aeruginosa* strains was picked up and pre-cultivated (ca. 16 h) at 37 °C with shacking (160 rpm) in Luria Bertani medium (hereinafter named as LB and composed of (g L^−1^): sodium chloride, 10; yeast extract, 5; tryptone, 10). The same medium was solidified by adding 15 g L^−1^ of bacteriological agar when needed. Bacterial cells were then inoculated (1% *v*/*v*) in LB medium amended with increasing concentrations—ranging from 0 to 100 μM as initial concentration—of each pyrrolomycin tested, challenging the cultures for 24 h and incubating them as reported above. The actual number of viable colony forming units per milliliter of culture (CFU mL^−1^) of each bacterial strain, who survived the challenge exerted by pyrrolomycins, was evaluated through the spot plate count method and compared to unchallenged cultures, as reported elsewhere [40,41,42]. The data are reported as average values (*n* = 3) of CFU mL^−1^ in the logarithmic (Log_10_) scale with standard deviation (SD). Evaluation of the MIC of PM-**5c**, and -**5d** against *S. aureus* and of PM-**5d** against *P. aeruginosa* was performed by incubating 1% (*v*/*v*) of an overnight bacterial suspension in LB medium in presence of increasing concentrations of the PMs. As a control, MIC of PM-**C** was measured. At the time of the inoculum and after overnight challenge, the absorbance of the cultures at 600 nm, which is an indirect way to measure the bacterial cell growth, was registered using a Spark™ 10 M multimode microplate reader measuring. The experiment was carried out in triplicate, being the absorbance corrected by subtracting that of the culture medium amended with the corresponding PM tested.

#### 4.2.2. Cell Cultures and Treatments

The HCT116 colon cancer cell line (ATCC^®^ CCL-247^™^), the MCF 7 breast cancer cell line (ATCC^®^ HTB-22™) and the hTERT RPE-1 -immortalized retinal pigment epithelial cell line (ATCC^®^ CRL-4000™) used in this study were cultured in DMEM medium (Gibco, Paisley, UK), supplemented with 1% glutamine, 10% heat-inactivated fetal bovine serum and 100U/mL penicillin and 100 μg/mL streptomycin, as already described [43,44,45,46], and maintained at 37 °C and 5% CO_2_. 

#### 4.2.3. MTT Viability Assay

Cells were plated at 5 × 10^3^ cells/well in 96-well plates and incubated for 24 h prior to treatment for further 48 h of different concentrations of PMs. After incubation, a sample of 20 μL of Thiazolyl Blue Tetrazolium Bromide (5 mg mL^−1^) in PBS was added to each well and cultured for 2h at 37 °C in CO_2_ incubator. After removing the medium containing MTT and three washes with PBS, 100 μL of warm DMSO was added to each well to dissolve formazan. Samples were shaken for 10 min, at room temperature in the dark, and the absorbance was recorded at 570 nm using a microplate reader (Spark^®^ 20M Tecan Trading AG, Switzerland). Percentage of cell viability with respect to untreated control cells (vehicle control) was calculated after subtraction of the blank. The IC_50_ values, that is the concentration necessary to inhibit the 50% of cell growth, was calculated using a dose-response model, obtained from sigmoidal fitting of response curves of percent inhibition versus logarithmic concentration of PMs, using Graph Pad Prism software. Each result represents the mean value of three different experiments performed in triplicate. 

#### 4.2.4. Morphological Assessment and Acridine Orange/Ethidium Bromide (AO/EB) Staining

HCT116 cells were seeded in 24 well culture plates with a cell density of 1 × 10^5^ cells/well. After 24 h cells were incubated for 24 h with different concentration of the selected PMs, and observed under a phase contrast inverted microscope (Carl Zeiss) at 200× magnification, for morphological changes assessment.

For AO/EB, the cells were seeded at the same conditions in a cover slip and after 24h and 48 of IC_50_ treatments, cells were washed twice with PBS and stained for few minutes with 200 μL of the Acridine Orange (100 μg/mL), Ethidium Bromide (100 μg/mL) mixture (1:1, *v*/*v*). Cells were immediately observed under a fluorescent microscopy (Carl Zeiss) at 630 × magnification.

## Figures and Tables

**Table 1:** Antibacterial activity and mammalian cell cytotoxicity of PMs (expressed in μM) analysed in this study. In bold the best-obtained values of minimal bactericidal concentration (MBC), IC_50_, and selectivity indexes versus cancer cells or bacteria.

PMs	MBC ^a^ *P. aeruginosa*	MBC ^a^ *S. aureus*	IC_50_ *HCT116* ^b^	IC_50_ *MCF 7* ^b^	IC_50_ *hTERT-RPE-1* ^b^	C-SI ^c^	B-SI ^d^
**C**	>100	90	**0.8 ± 0.3**	**1.5 ± 0.4**	8.3 ± 1.9	10.4–5.5	>0.01–0.9
**1**	>100	**5**	**1.3 ± 0.3**	**1.2 ± 0.7**	57.7 ± 11.2	**44.2-47.3**	>0.6–**11.5**
**2**	>100	70	11.1 ± 3.3	17.2 ± 3.2	149 ± 41	**13.4-8.6**	>1.5–2.1
**5a**	>100	60	**1.9 ± 0.42**	**2.2 ± 0.3**	65.8 ± 10.5	**34.6–29.2**	>0.7-1.1
**5b**	>100	20	18.7 ± 3.8	28.8 ±4.2	55.4 ± 13.2	3–1.9	>0.6–2.8
**5c**	>100	1	7.6 ± 1.9	12.0± 2.8	29.7 ± 5.7	10.4–5.5	>0.3–**29.7**
**5d**	30	**7.5**	**1.6 ± 0.2**	**1.6 ± 0.4**	224.9 ± 22.7	**143.3–143.4**	**7.5–30**

^a^ MBC: Minimum Bactericidal Concentration of PMs against *P. aeruginosa* and *S. aureus*. ^b^ IC_50_: Concentration of the PMs at which the proliferation of cancer HCT116, MCF 7 and the normal hTERT-RPE-1 cells was inhibited by 50% with respect to the untreated growth control after 48 h of treatment. ^c^ CSI: Cancer Selectivity Index was defined as ratio of IC_50_ of normal hTERT-RPE-1 to that of HCT116 and MCF 7 cancer cells, respectively. ^d^ BSI: Bacterial Selectivity Index was defined as ratio of IC_50_ of normal hTERT-RPE-1 to minimal bactericidal concentration (MBC) of *P. aeruginosa* and *S. aureus*, respectively.

**Table 2:** Calculated ADMET (Absorption, Distribution, Metabolism, Elimination and Toxicity) properties of PM-**C**, **1**, **2**, and **5a-d**.

Heading	PM-C	1	2	5a	5b	5c	5d	Range 95% of Drugs
cLogPo/w	4.476	4.889	5.637	2.496	3.200	3.132	3.746	−2.0/6.5
cLogS	−5.031	−6.250	−6.331	−4.126	−4.744	−4.945	−5.419	−6.5/0.5
cLogKhsa	0.364	0.568	0.628	0.108	0.209	0.240	0.319	−1.5/1.5
cLogBB	0.219	0.376	1.039	−1.138	−0.681	−0.890	−0.444	−3.0/1.2
CNS Activity	+	+	++	− −	+/−	−	+/−	− − (inactive) ++ (active)
cLogHERG	−4.511	−4.436	−4.259	−4.586	−4.583	−4.627	−4.515	concern below −5
cPCaco	1298	1338	7162	131	277	175	344	<25 poor >500 great
clogKp	−2.518	−2.594	−1.215	−4.245	−3.667	−4.111	−3.617	Kp in cm/hr
Percent Human Oral Absorption ±20%	100	100	100	79	89	85	94	<25% poor
Jorgensen Rule	0	1	1	0	0	0	0	maximum 3
Lipinski Rule	0	1	2	0	0	0	0	maximum 4
# Stars (violation of the 95% range)	2	2	2	0	1	1	1	0-5

cLogPo/w: predicted octanol/water partition coefficient. cLogS: predicted aqueous solubility, log S. S in mol dm^−3^ is the concentration of the solute in a saturated solution that is in equilibrium with the crystalline solid. cLogKhsa: prediction of binding to human serum albumin. cLogBB: predicted brain/blood partition coefficient. Note: Predictions are for orally delivered drugs. CNS Activity: predicted central nervous system activity on a−2 (inactive) to +2 (active) scale. cLogHERG: predicted IC_50_ value for blockage of HERG K^+^ channels. cPCaco: Predicted apparent Caco-2 cell permeability in nm/sec; Caco-2 cells are a model for the gut-blood barrier. Predictions are for non-active transport. cLogKp: predicted skin permeability, log *K*. Percent Human-Oral Absorption ±20%: predicted human oral absorption on 0 to 100% scale; the prediction is based on a quantitative multiple linear regression model; the assessment uses a knowledge-based set of rules, including number of metabolites, number of rotatable bonds, logP, solubility, and cell permeability. Jorgensen Rule or Rule of Three: number of violations of Jorgensen’s rule of three. The three rules are: cLogS > −5.7, cPCaco > 22 nm/s, # Primary Metabolites < 7. Compounds with fewer (and preferably no) violations of these rules are more likely to be orally available. Lipinski Rule or Rule of Five: number of violations of Lipinski’s rule of five. The four rules are: mol_MW < 500, cLogPo/w < 5, HB donor ≤ 5, HB acceptor ≤ 10. Compounds that satisfy these rules are considered drug-like. # Stars (violation of the 95% range): number of property or descriptor values that are outside the 95% range of similar values for known drugs. A large number of stars suggests that a molecule is less drug-like than molecules with few stars.

## Supplementary Materials

The following are available online at https://www.mdpi.com/2079-6382/9/6/292/s1, Figure S1: NMR spectra of new pyrrolomycins **2** and **5a**–**d**. Table S1: MIC and MBC (expressed in μM) of PM-**5c**, -**5d** and -**C**. Table S2: ADMET properties of the pyrrolomycins **C**, **1**, **2** and **5a**–**d** calculated by QikProp software v6.2. Table S3: ADMET properties of the pyrrolomycins **C**, **1**, **2** and **5a**–**d** calculated by pkCSM–pharmacokinetics.
