# Supplementary material for: New Synthetic Nitro-Pyrrolomycins as Promising Antibacterial and Anticancer Agents
Source: Antibiotics (Basel). 2020 May 30;9(6):292. doi: 10.3390/antibiotics9060292 (PMC7345095; doi:10.3390/antibiotics9060292)
Supplement: Supplementary file 1 [file antibiotics-09-00292-s001.pdf]

New synthetic nitro-pyrrolomycins as promising antibacterial and anticancer agents

Maria Valeria Raimondi [1,†], Alessandro Presentato [2,†], Giovanna Li Petri [1,*], Miriam Buttacavoli [2], Agnese Ribaudo [1,3], Viviana De Caro [1], Rosa Alduina [2,*] and Patrizia Cancemi [2]

[1] Department of Biological, Chemical and Pharmaceutical Sciences and Technologies (STEBICEF), University of Palermo, via Archirafi 32, 90123 Palermo, Italy; mariavaleria.raimondi@unipa.it (M.V.R.); giovanna.lipetri@unipa.it (G.L.P.); viviana.decaro@unipa.it (V.D.C)

[2] Department of Biological, Chemical and Pharmaceutical Sciences and Technologies (STEBICEF), University of Palermo, viale delle Scienze, Building 16, 90128 Palermo, Italy; alessandro.presentato@unipa.it (A.P.); miriam.buttacavoli@unipa.it (M.B.); valeria.alduina@unipa.it (R.A.); patrizia.cancemi@unipa.it (P.C.)

[3] Pharmaceutical Department, Provincial Health Authority (ASP) of Palermo, via Pindemonte 88, 90129 Palermo, Italy; agnese.ribaudo@community.unipa.it (A.R.)

* Correspondence: giovanna.lipetri@unipa.it (G.L.P.); valeria.alduina@unipa.it (R.A.)

† These authors contributed equally to the work.

Figure S1. NMR spectra of new pyrrolomycins **2** and **5a-d.**

^{1}H-NMR of (3,5-dibromo-2-methoxyphenyl)(3,4,5-tribromo-1-methyl-1*H*-pyrrol-2-yl)methanone (**2**)

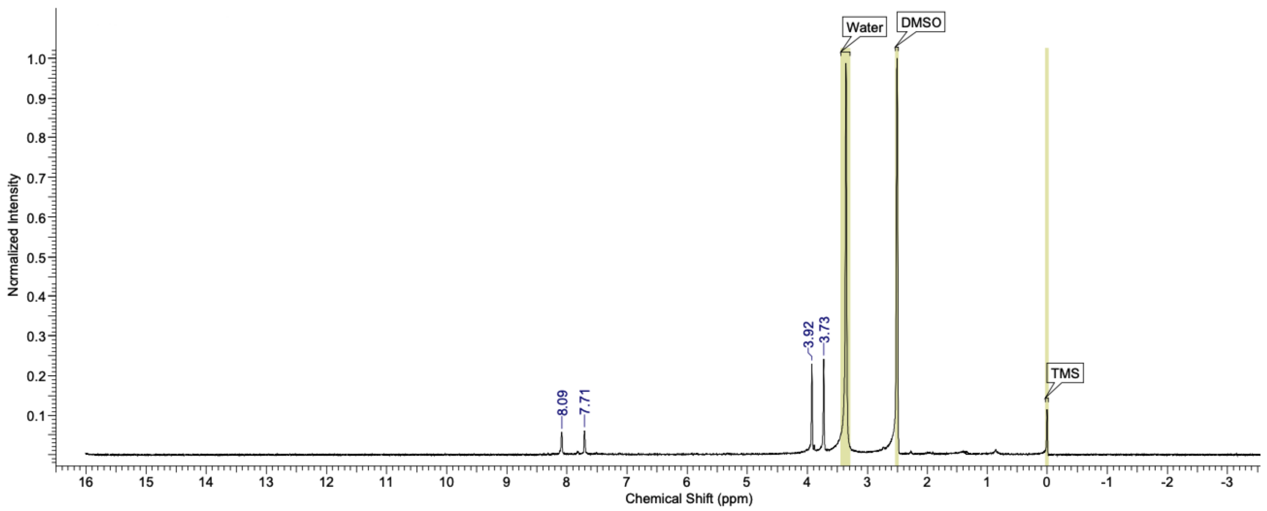

^{13}C-NMR of (3,5-dibromo-2-methoxyphenyl)(3,4,5-tribromo-1-methyl-1*H*-pyrrol-2-yl)methanone (**2**)

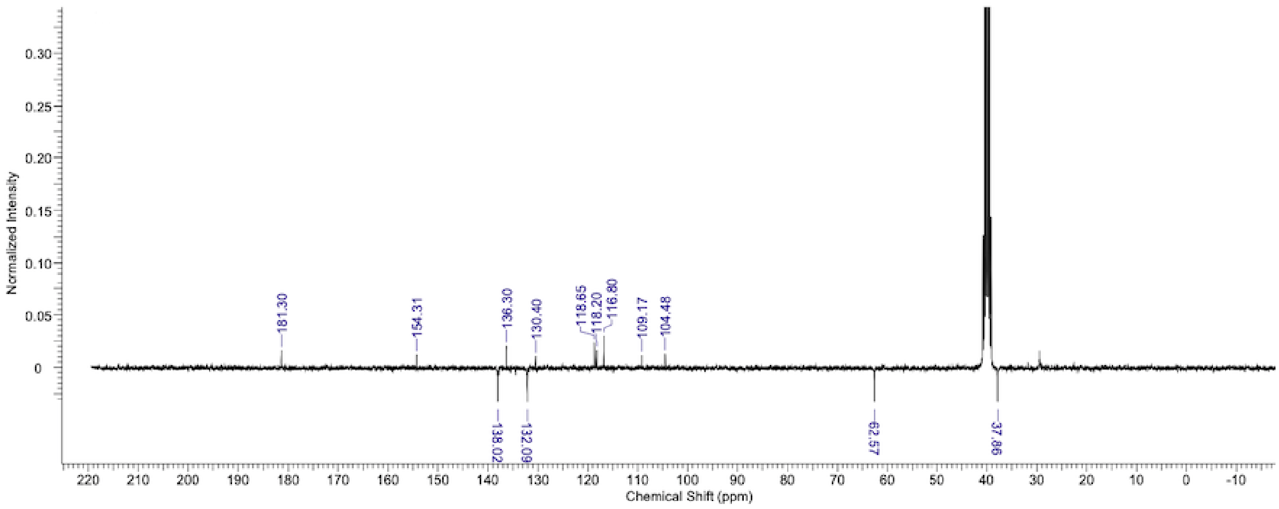

^{1}H-NMR of (3,5-dichloro-2-hydroxyphenyl)(4-nitro-1*H*-pyrrol-2-yl)methanone (**5a**)

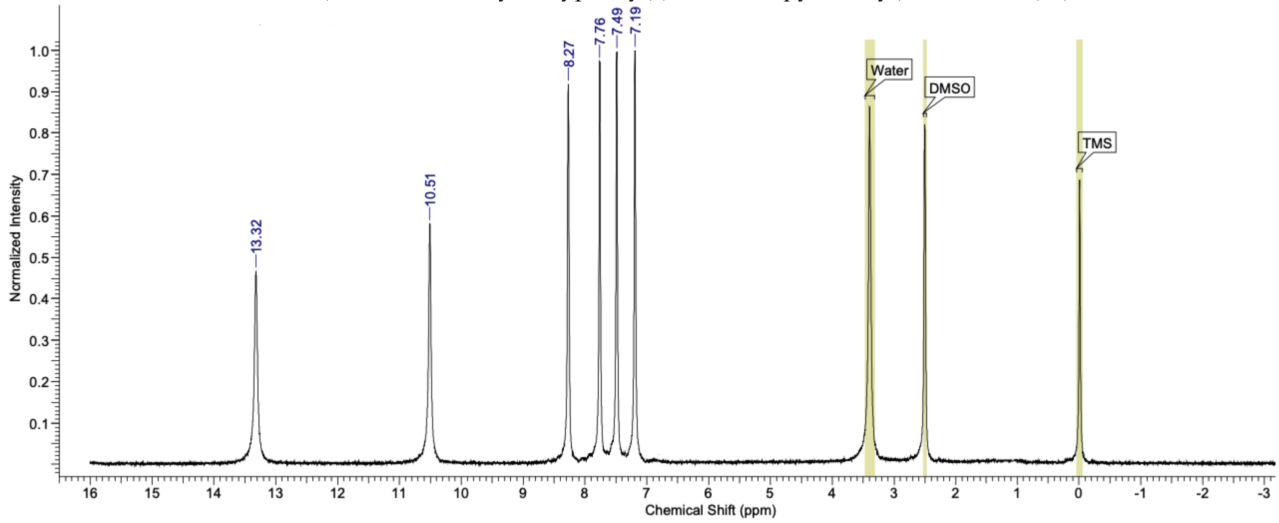

^{13}C-NMR of (3,5-dichloro-2-hydroxyphenyl)(4-nitro-1*H*-pyrrol-2-yl)methanone (**5a**)

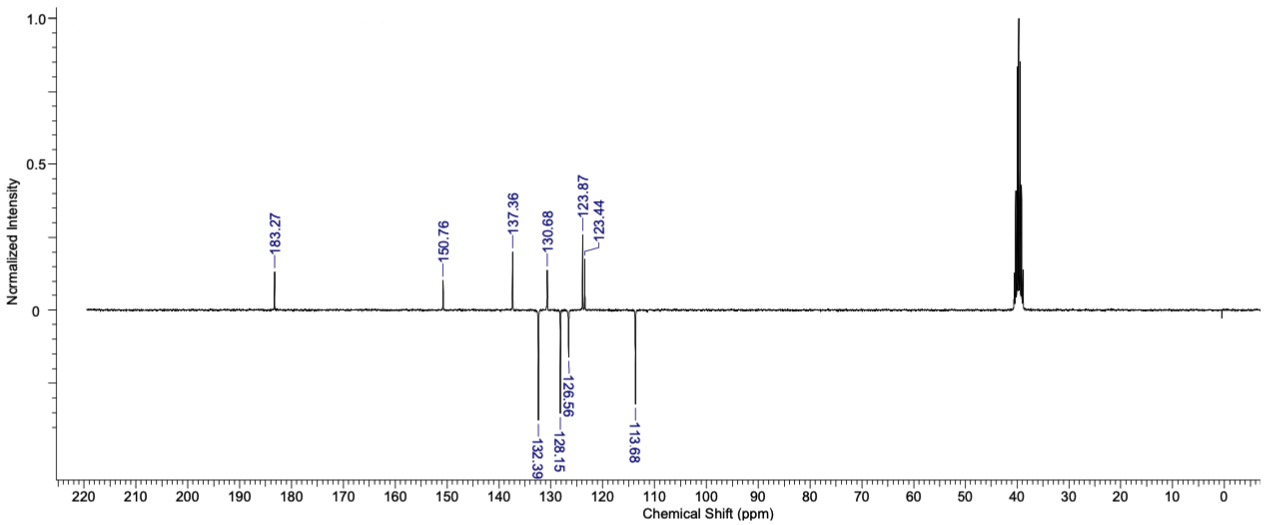

^{1}H-NMR of (4-chloro-5-nitro-1*H*-pyrrol-2-yl)(3,5-dichloro-2-hydroxyphenyl)methanone (**5b**)

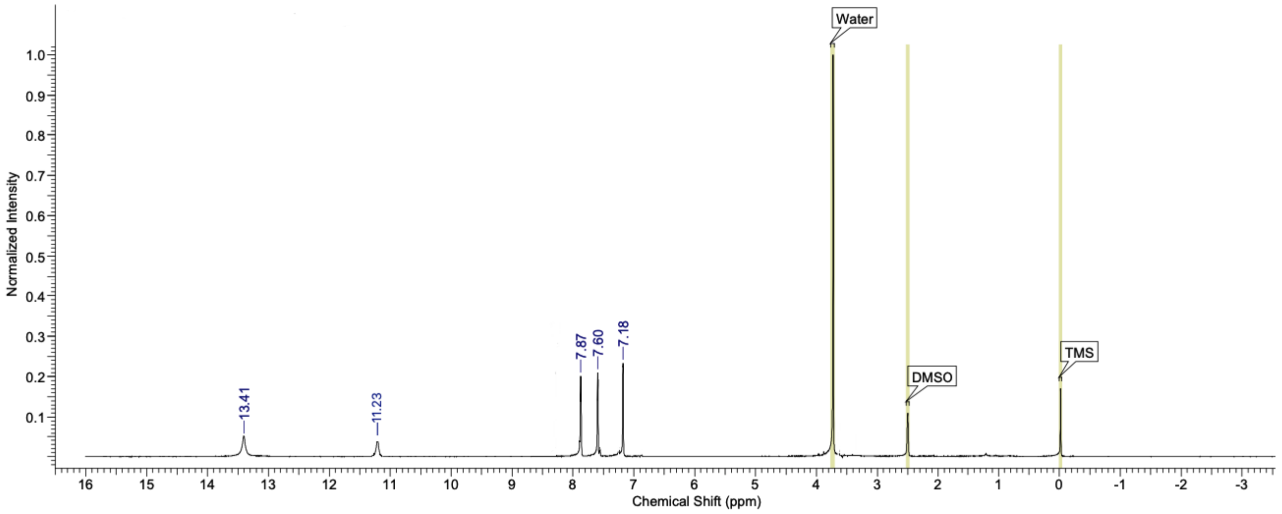

^{13}C-NMR of (4-chloro-5-nitro-1*H*-pyrrol-2-yl)(3,5-dichloro-2-hydroxyphenyl)methanone (**5b**)

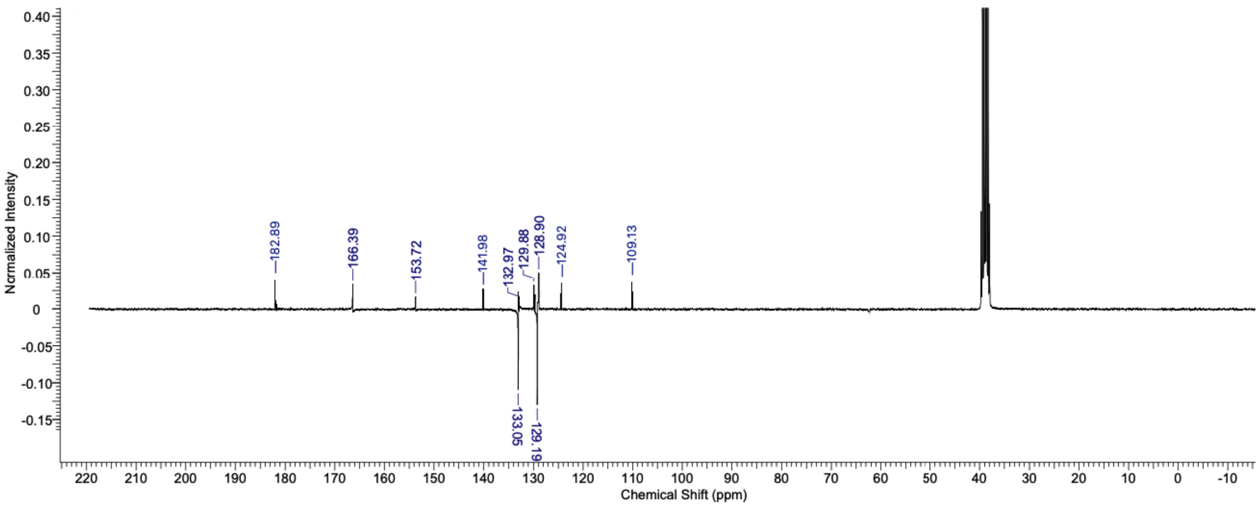

^{1}H-NMR of (4-bromo-5-nitro-1*H*-pyrrol-2-yl)(3,5-dichloro-2-hydroxyphenyl)methanone (**5c**)

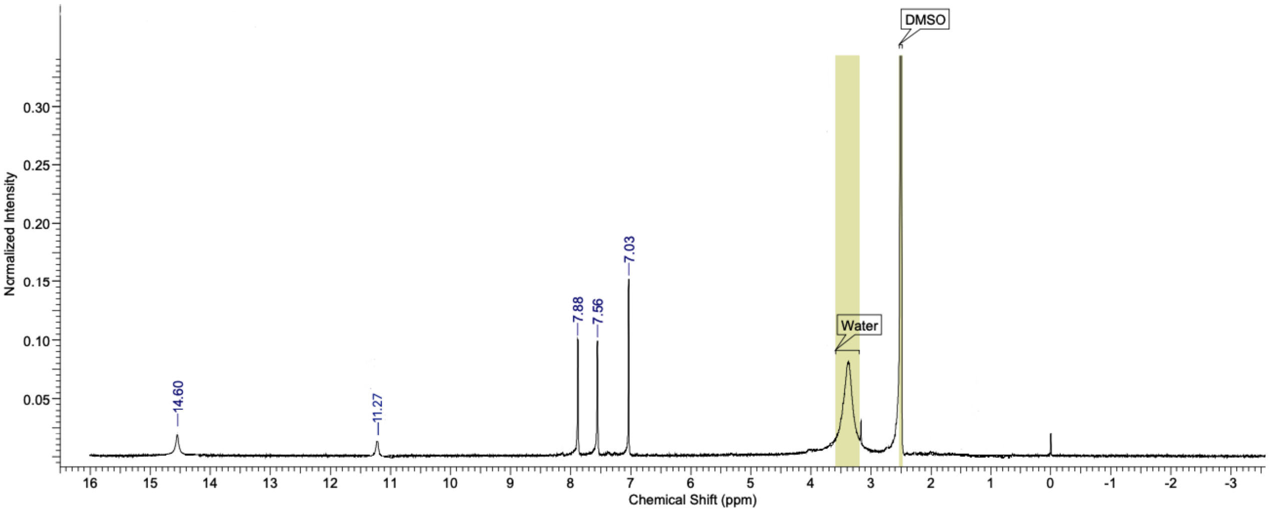

^{13}C-NMR of (4-bromo-5-nitro-1*H*-pyrrol-2-yl)(3,5-dichloro-2-hydroxyphenyl)methanone (**5c**)

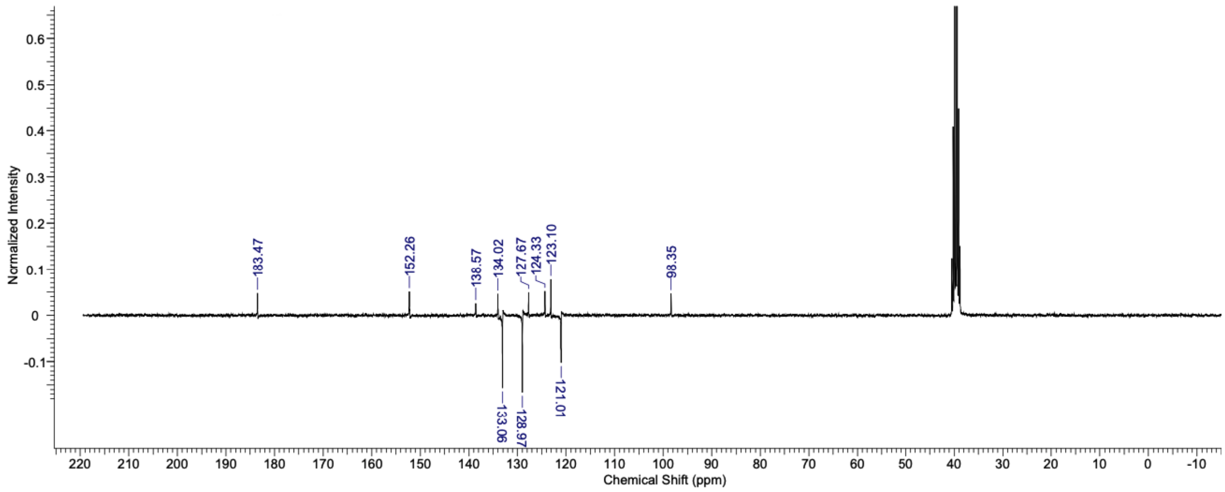

^{1}H-NMR of (3,5-dichloro-2-hydroxyphenyl)(4,5-dichloro-3-nitro-1*H*-pyrrol-2-yl)methanone (**5d**)

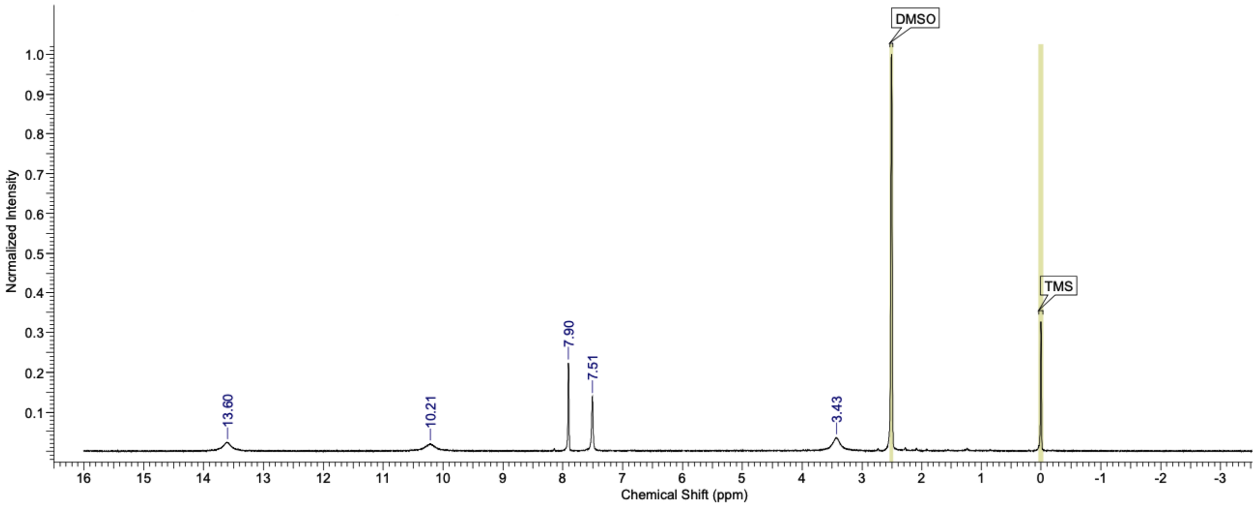

^{13}C-NMR of (3,5-dichloro-2-hydroxyphenyl)(4,5-dichloro-3-nitro-1*H*-pyrrol-2-yl)methanone (**5d**)

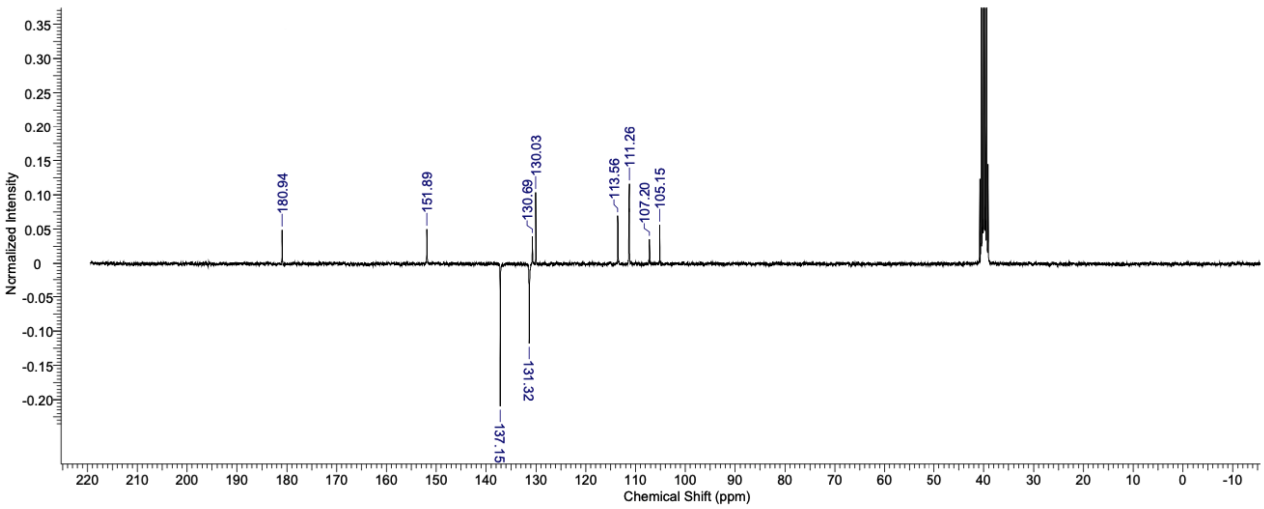

Table S1. MIC and MBC (expressed in μM) of PM-5c, -5d and -C.

PMs	MIC (μM)	MBC (μM)
Staphylococcus aureus		
5c	0.5	1
5d	2	7.5
PM-C	1	90
Pseudomonas aeruginosa		
5d	>20	30
PM-C	>40	>100

Table S2. ADMET properties of the pyrrolomycins C, **1**, **2** and **5a-d** calculated by QikProp software v6.2.

	C	1	2	5a	5b	5c	5d	Range 95% of Drugs
Primary metabolites & Reactive functional groups	Metabolism likely: aromatic OH oxidation	Metabolism likely: aromatic OH oxidation	Metabolism likely: ether dealkylation	Metabolism likely: aromatic OH oxidation	Metabolism likely: aromatic OH oxidation	Metabolism likely: aromatic OH oxidation	Metabolism likely: aromatic OH oxidation	
<u>Principal Descriptors</u>								
MW	324.978	581.678	609.732	301.085	335.530	379.981	369.976	130.0 / 725.0
Dipole Moment (D)	1.254	1.867	2.701	4.980	5.428	6.658	11.344	1.0 / 12.5
Total SASA	496.448	528.172	561.220	488.735	506.239	515.745	526.880	300.0 / 1000.0
Hydrophobic SASA	0.000	0.000	141.227	0.000	0.000	0.000	0.000	0.0 / 750.0
Hydrophilic SASA	93.048	91.667	14.850	197.867	163.803	184.674	153.812	7.0 / 330.0
Carbon Pi SASA	121.461	92.720	82.449	152.468	138.433	121.469	100.342	0.0 / 450.0
Weakly Polar SASA	281.939	343.785	322.694	138.400	204.002	209.602	272.726	0.0 / 175.0
Molecular Volume (A^3)	817.470	891.061	989.632	804.835	842.621	854.696	883.605	500.0 /2000.0
vdW Polar SA (PSA)	57.549	57.881	30.538	103.739	97.854	101.840	97.648	7.0 / 200.0
No. of Rotatable Bonds	3.000	3.000	3.000	4.000	4.000	4.000	4.000	0.0 / 15.0
HB Donor	1.000	1.000	0.000	1.000	1.000	1.000	1.000	0.0 / 6.0
HB Acceptor	1.750	1.750	2.750	2.750	2.750	2.750	2.750	2.0 / 20.0
Globularity (Sphere = 1)	0.852	0.848	0.856	0.856	0.852	0.845	0.845	0.75 / 0.95
Ionization Potential (eV)	9.136	9.631	9.579	9.549	9.755	9.641	9.682	7.9 / 10.5
Electron Affinity (eV)	0.765	1.030	1.083	1.029	1.472	1.477	1.389	-0.9 / 1.7
<u>Properties Predictions</u>								
Polarizability (A^3)	25.795	28.465	32.312	24.943	26.321	26.643	27.598	13.0 / 70.0
cLogPC16	9.423	10.376	10.384	9.263	9.752	9.906	10.281	4.0 / 18.0

cLogPoct	11.702	12.919	12.945	12.175	12.843	13.270	15.023	8.0 / 35.0
cLogPw	4.998	4.891	3.892	6.618	6.356	6.407	6.102	4.0 / 45.0
cLogPo/w	4.476	4.889	5.637	2.496	3.200	3.132	3.746	-2.0 / 6.5
cLogS	-5.031	-6.250		-4.126	-4.744	-4.945	-5.419	-6.5 / 0.5
CIcLogS	-5.748	-11.141	-6.331	-4.869	-5.577	-6.498	-6.290	-6.5 / 0.5
cLogKhsa	0.364	0.568	-11.405	0.108	0.209	0.240	0.319	-1.5 / 1.5
cLogBB	0.219	0.376	0.628	-1.138	-0.681	-0.890	-0.444	-3.0 / 1.2
No. of Primary Metabolites	1	1	1.039	1	1	1	1	1.0 / 8.0
CNS Activity	+	+	1	- -	+/-	-	+/-	- - (inactive) ++ (active)
cLogHERG	-4.511	-4.436	++	-4.586	-4.583	-4.627	-4.515	concern below -5
cPCaco	1298	1338	-4.259	131	277	175	344	< 25 poor > 500 great
cPMDCK	10000	10000	7162	316	1619	1061	4877	< 25 poor > 500 great
clogKp	-2.518	-2.594	10000	-4.245	-3.667	-4.111	-3.617	Kp in cm/hr
Jm, max transdermal transport rate	0.004	0.001	-1.215	0.001	0.001	0.000	0.000	micrograms/cm^2-hr
Percent Human Oral Absorption ±20%	100	100	100	79	89	85	94	< 25% poor
Jorgensen Rule	0	1	1	0	0	0	0	maximum 3
Lipinski Rule	0	1	2	0	0	0	0	maximum 4
Qual. Model for Human Oral Absorption	High	Low	Low	High	High	High	High	>80% is high
# Stars (violation of the 95% range)	2	2	2	0	1	1	1	0-5

Principal Descriptors. MW: molecular weight of the molecule. **Dipole Moment (D)**: computed dipole moment of the molecule. **Total SASA**: total solvent accessible surface area (SASA) in square angstroms using a probe with a 1.4 Å radius. **Hydrophobic SASA**: hydrophobic component of the SASA (saturated carbon and attached hydrogen). **Hydrophilic SASA**: hydrophilic component of the SASA (SASA on N, O, and H on heteroatoms). **Carbon Pi SASA**: (carbon and attached hydrogen) component of the SASA. **Weakly Polar SASA**: weakly polar component of the SASA (halogens, P, and S). **Molecular Volume (A^3)**: total solvent-accessible volume in cubic angstroms using a probe with a 1.4 Å radius. **vdW Polar SA (PSA)**: Van der Waals surface area of polar nitrogen and oxygen atoms. **No. of Rotatable Bonds**: estimated number of rotatable bonds that could influence interaction with the biological substrate. **HB Donor**: estimated number of hydrogen bonds that would be donated by the solute to water molecules in an aqueous solution. Values are averages taken over a number of configurations, so they can be non-integer. **HB Acceptor**: estimated number of hydrogen bonds that would be accepted by the solute from water molecules in an aqueous solution. Values are averages taken over a number of configurations, so they can be non-integer. **Globularity (Sphere = 1)**: globularity descriptor, $(4\pi r^2)/(SASA)$, where r is the radius of a sphere with a volume equal to the molecular volume. Globularity is 1.0 for a spherical molecule. **Ionization Potential (eV)**: PM3 calculated ionization potential. **Electron Affinity (eV)**: PM3 calculated electron affinity.

Properties Predictions. Polarizability (A^3): predicted polarizability in cubic angstroms. **cLogPC16**: predicted hexadecane/gas partition coefficient. **cLogPoct**: predicted octanol/gas partition coefficient. **cLogPw**: predicted water/gas partition coefficient. **cLogPo/w**: predicted octanol/water partition coefficient. **cLogS**: predicted aqueous solubility, log S. S in mol dm^{-3} is the concentration of the solute in a saturated solution that is in equilibrium with the crystalline solid. **CIcLogS**: conformation-independent predicted aqueous solubility, log S; S in mol dm^{-3} is the concentration of the solute in a saturated solution that is in equilibrium with the crystalline solid. **cLogKhsa**: prediction of binding to human serum albumin. **cLogBB**: predicted brain/blood partition coefficient. Note: Predictions are for orally delivered drugs. **No. of Primary Metabolites**: Number of likely metabolic reactions. **CNS Activity**: predicted central nervous system activity on a –2 (inactive) to +2 (active) scale. **cLogHERG**: predicted IC_{50} value for blockage of HERG K^+ channels. **cPCaco**: Predicted

apparent Caco-2 cell permeability in nm/sec; Caco-2 cells are a model for the gut-blood barrier. Predictions are for non-active transport. **cPMDCK**: predicted apparent MDCK cell permeability in nm/sec. MDCK cells are considered to be a good mimic for the blood-brain barrier. Predictions are for non-active transport. **cLogKp**: predicted skin permeability, log *K*. **Jm, max transdermal transport rate**: predicted maximum transdermal transport rate. **Percent Human-Oral Absorption ±20%**: predicted human oral absorption on 0 to 100% scale; the prediction is based on a quantitative multiple linear regression model; the assessment uses a knowledge-based set of rules, including number of metabolites, number of rotatable bonds, logP, solubility and cell permeability. **Jorgensen Rule or Rule of Three**: number of violations of Jorgensen's rule of three. The three rules are: cLogS > -5.7, cPCaco > 22 nm/s, # Primary Metabolites < 7. Compounds with fewer (and preferably no) violations of these rules are more likely to be orally available. **Lipinski Rule or Rule of Five**: number of violations of Lipinski's rule of five. The four rules are: mol_MW < 500, cLogPo/w < 5, HB donor ≤ 5, HB acceptor ≤ 10. Compounds that satisfy these rules are considered drug-like. **Qual. Model for Human Oral Absorption**: predicted qualitative human oral absorption: 1, 2, or 3 for low, medium, or high. #**Stars (violation of the 95% range)**: number of property or descriptor values that are outside the 95% range of similar values for known drugs. A large number of stars suggests that a molecule is less drug-like than molecules with few stars.

Table S3. ADMET properties of the pyrrolomycins C, **1**, **2** and **5a-d** calculated by pkCSM – pharmacokinetics.

Properties Predictions	C	1	2	5a	5b	5c	5d	
VDss (human)	0.076	0.119	0.159	-0.138	-0.041	-0.025	-0.156	*Low < -0.15; High > 0.45 log L/kg*
Total Clearance	0.386	-0.252	-0.213	0.51	0.207	0.140	0.313	*log ml/min/kg*
Renal OCT2 substrate	No	No	No	No	No	No	Yes	*Yes/No*
CYP2D6 substrate	No	No	No	No	No	No	No	*Yes/No*
CYP3A4 substrate	No	Yes	Yes	No	No	No	Yes	*Yes/No*
CYP1A2 inhibitor	Yes	Yes	Yes	Yes	Yes	Yes	Yes	*Yes/No*
CYP2C19 inhibitor	Yes	Yes	Yes	Yes	Yes	Yes	Yes	*Yes/No*
CYP2C9 inhibitor	No	Yes	Yes	No	No	No	No	*Yes/No*
CYP2D6 inhibitor	No	No	No	No	No	No	No	*Yes/No*
CYP3A4 inhibitor	No	Yes	No	No	No	No	No	*Yes/No*

Properties Predictions. VDss: predicted steady state volume of distribution in human. **Total Clearance**: predicted drug clearance as a combination of hepatic and renal clearance. **Renal OCT2 substrate**: prediction of binding to Organic Cation Transporter 2. **CYP2D6/CYP3A4 substrate**: prediction of binding to CYP2D6/CYP3A4. **CYP1A2/CYP2C19/CYP2D6/ CYP3A4 inhibitors**: 1A2/2C19/2D6/3A4 inhibition predictions.
